# Knowledge categorization affects popularity and quality of Wikipedia articles

**DOI:** 10.1371/journal.pone.0190674

**Published:** 2018-01-02

**Authors:** Jürgen Lerner, Alessandro Lomi

**Affiliations:** 1 University of Konstanz, Konstanz, Germany; 2 Università della Svizzera italiana, Lugano, Switzerland; 3 University of Southern California, Los Angeles, California, United States of America; Aarhus Universitet, DENMARK

## Abstract

The existence of a shared classification system is essential to knowledge production, transfer, and sharing. Studies of knowledge classification, however, rarely consider the fact that knowledge categories exist within hierarchical information systems designed to facilitate knowledge search and discovery. This neglect is problematic whenever information about categorical membership is itself used to evaluate the quality of the items that the category contains. The main objective of this paper is to show that the effects of category membership depend on the position that a category occupies in the hierarchical knowledge classification system of Wikipedia—an open knowledge production and sharing platform taking the form of a freely accessible on-line encyclopedia. Using data on all English-language Wikipedia articles, we examine how the position that a category occupies in the classification hierarchy affects the attention that articles in that category attract from Wikipedia editors, and their evaluation of quality of the Wikipedia articles. Specifically, we show that Wikipedia articles assigned to coarse-grained categories (i. e., categories that occupy higher positions in the hierarchical knowledge classification system) garner more attention from Wikipedia editors (i. e., attract a higher volume of text editing activity), but receive lower evaluations (i. e., they are considered to be of lower quality). The negative relation between attention and quality implied by this result is consistent with current theories of social categorization, but it also goes beyond available results by showing that the effects of categorization on evaluation depend on the position that a category occupies in a hierarchical knowledge classification system.

## Introduction

Social categories give order to past experiences and shape expectations about the future. Categories matter because they provide labels that are used to define the identity of objects [1, 2], and adjust individual behavior to social situations—a view widely shared across studies of products, individuals, and institutions [3–5]. Categories matter also because the aggregate information they convey helps to predict features of individual items they contain [6]. As a consequence, categorization drives a number of day-to-day decisions such as, for example, what restaurant to try, what movie to watch, or what stock to buy [7–9].

In this paper we contribute to this active line of interdisciplinary research by focusing our attention on knowledge categories—sets of labels commonly used to partition and organize knowledge spaces into coherent thematic sub-domains. More specifically, we analyze how categorical identities affect the production and evaluation of encyclopedic articles in Wikipedia, the “free encyclopedia that anyone can edit” (en.wikipedia.org). Building on prior work [10], we define the *granularity* of a category as a function of its position in a hierarchical system of classification. A Wikipedia knowledge category is coarse-grained (fine-grained) to the extent that it is close to (far from) its root category. In other words, a knowledge category is coarse-grained (fine-grained) to the extent that it is located high (low) in the Wikipedia knowledge hierarchy consisting of more that 1,000,000 categories. Following [10], we rely on graph-theoretic properties of hierarchical category systems and define the granularity of a category as the shortest path distance from its root category. We go beyond existing research by specifying this construct in the context of a model that predicts quality of a Wikipedia article in terms of the granularity of the knowledge categories in which the article is included.

More specifically, we argue and demonstrate that the granularity of a category influences the amount of contributions that articles within that category will be able to attract from Wikipedia editors. We also show that the granularity of a category affects the probability that articles in that category will be included in the restricted set of high quality Wikipedia articles, called *featured articles*. Since the purpose of Wikipedia as a knowledge production and organization system is to attract voluntary contributions to create a high-quality encyclopedia, it is hard to think of a more important and vital set of questions than those pertaining to the factors that regulate the productivity and engagement of Wikipedia editors, and the quality of their contribution. We report clear evidence that articles in coarse-grained categories are more likely to be popular, but less likely to be featured. These results demonstrate the powerful effect that categorization has on the use and evaluation of knowledge [11]. While the scope of the empirical results we present could be considered somewhat specific to Wikipedia as a particular hierarchical knowledge system, Wikipedia is also the 5th most visited site in the world [12]. As such Wikipedia is not only globally adopted and recognized, but also highly scientifically relevant [11] and broadly representative of a new generation of decentralized on-line production systems providing public goods on a voluntary basis [13, 14].

After this general introduction, the article is organized as follows. In the next section we discuss the theoretical background that inspires and guides our interest in Wikipedia as a valuable empirical setting to study the effect of knowledge categorization on the popularity and social evaluation of knowledge items. We argue that information science has produced important insights that can be brought to bear on a more general sociological understanding of Wikipedia as a concrete example of a social classification system. In this sense, it is important to observe that the contribution of our work is not to information science, but to organizational sociology. In the subsequent section we outline the essential elements of our empirical context. We then describe the data and the analytical approach that we implement and report the results of the study. A discussion section concludes the article by offering possible interpretations of the empirical results in the light of our theoretical narrative.

## Theoretical background

Categorical identity—the set of qualities collectively attributed to an object by virtue of its membership in socially accepted categories—plays a central role in the organization and evaluation of knowledge [15]. This may happen, for example, because categories store information that may be used to estimate the value (or utility) of the features that category members share [6]. In consequence, a category that helps accurately to predict the features of the objects it contains, is more likely to be considered useful than a category with lower predictive value. This statement summarizes the so called category utility hypothesis that is central in the cognitive psychology of categorization [16].

Categorization is not only a cognitive, but also a social process [9]. As such, categorization frequently happens to be the outcome of negotiation, contention, and competition among rival views. Consider, for example, the so called *Pluto controversy* triggered by the decision of the International Astronomical Union (IAU) to re-define “planet” in a way that excludes Pluto from the category of “planets” [17]. According to the new classification criteria established by the IAU Pluto is now a “Trans-Neptunian” object and, at most, a “dwarf planet.” Some astronomers consider the IAU’s classification criteria “flawed” because—if applied strictly—they would also exclude Earth, Mars, Jupiter and Neptune from full membership in the category of “planets” [18]. If accepted, the new classification would have major implications for the way we currently understand the solar system. Clearly, in this case cognition explains only part of the classification process.

Recognizing the social character of categorization, a major line of research in organizational sociology, has called attention on the relation between categorical identity and collective evaluation processes [19, 20]. According to this line of research, the value assigned to an object by virtue of category membership depends on a process of collective evaluation performed by relevant audiences [5, 21]. Feature films, restaurants, and securities are only some of the empirical cases in which this theoretical insight has been shown to have consistent predictive value [7–9]. The process of central interest in these studies is not categorization per se, but rather the implication that membership in multiple categories has for social and economic evaluation.

The results of recent empirical research on categorical identities clearly resonate with core ideas in prototype theory originally proposed by Rosch [22–24]—one of the cornerstones of contemporary cognitive linguistics [25]. Current sociological research on categories and studies of categorization as the outcome of core cognitive processes share the view that membership in categories should be considered as graded (i. e., objects may be members in multiple categories), and that categorical boundaries are therefore permeable. Sociological and cognitive approaches differ in the importance they assign to the role played by collective consensus expressed by relevant audiences in determining both the antecedents as well as the consequences of category membership.

Despite the broad sociological interest in the antecedents and consequences of categorical identities, relatively little attention has been paid to the fact that categories are typically defined in terms of positions they occupy within complex hierarchical systems [26]. This lack of attention is surprising because the internal organizational structure of classification systems is known to affect a wide range of relevant outcomes such as individual search strategies, recall, discrimination, and fallout rates [27]. Furthermore, categorical identities may be considered as “ontological and lexical structures (that) are the underpinning of scientific and scholarly work, of learning, and of machine intelligence [28, p. 1 119].” These fundamental insights from information science have not yet been incorporated in a more general sociological understanding of the consequences of positions that knowledge objects occupy in a space of categories [5].

This gap is particularly noticeable in sociological works on analysis of Wikipedia where categories are nested within a tree-like hierarchical structure, and where most categories can be traced back to a small number of very broad categories [10]. By affecting the use and evaluation of knowledge, and the patterns of links among knowledge objects, the hierarchical position occupied by a category affects the level of attention that the items it contains will be likely to attract. But why would the position that a category occupies in the classification hierarchy affect the categorical identity of the items it contains—and hence the evaluation of the quality of those items? After all, should not the quality of an article in Wikipedia be dependent only on its intrinsic features?

In this article we demonstrate that one potentially useful way to address these questions involves the notion of category granularity. Coarse-grained categories occupy higher positions in the classification hierarchy. Fine-grained categories occupy lower positions, i. e., they are more distant from their root category. Building on prior work of [10] we compute the path distance of categories from their root and use it as a measure of granularity. As the grain of the categories increases (i. e., categories become narrower), categorical boundaries become sharper and less permeable. In consequence, objects have to satisfy stricter criteria in order to be admitted as category members. For this reason, objects in fine-grained categories receive a stronger identity from category membership and their qualities are easier to evaluate with reference to categorical boundaries [19]. The variance of opinions about the quality of objects included in high-grain categories is likely to be lower. Objects in coarse-grained categories are likely to attract more attention—i. e., to be appealing to broader audiences—but tend to attract less positive evaluations [7]. This happens because membership in coarse-grained categories conveys ambiguous identities that are unlikely to support convergent evaluations from relevant critical audiences [9].

As an illustrating example consider the two Wikipedia articles “Mathematics” and “Euclidean algorithm.” The former (“Mathematics”) belongs to very coarse categories and received 6,242 edits, which is an unusually high level of editing activity. However “Mathematics” is only rated B-class (the fourth-best in Wikipedia’s seven assessment grades). In contrast, the much more specific article “Euclidean algorithm” attracts a lower level of editing activity (2,150 edits), but is rated as a featured article—the highest quality label in Wikipedia.

This discrepancy between the level of editing activity that an article garners and its perceived quality that this example illustrates may be framed in terms of the so called principle of allocation proposed by Levins [29] and frequently invoked to explain differences in fitness (or “measure of success”) among social forms [7]. In brief, the principle of allocation posits a constant fitness that may be allocated across possible resource positions, or “niches.” This postulate implies a tradeoff between niche width and fitness levels. When applied to social forms, the principle of allocation predicts that forms spreading their fitness across multiple positions (generalists) will typically attain a lower level of fitness at each niche position than forms focusing more narrowly on one or few positions (specialists) [30].

Transposed to the specific case under discussion, the principle of allocation implies that the “fitness” of an article (the evaluation it receives from Wikipedia editors) may be affected by the granularity of the categories to which it belongs. Articles in coarse-grain categories (categories occupying higher positions in the global classification hierarchy) will attract a higher level of editing activity from a broader and larger set of editors—and hence have wider knowledge niches. This happens because editors without specialized knowledge, or with different background knowledge, may find it easier to edit articles contained in knowledge categories that are more broadly defined—and hence more lenient, or less constraining [31].

However, membership in coarse-grained categories will also make it more difficult to reach an agreement on the quality and appropriateness of articles contained in it because the fuzzy boundaries of the category increase the uncertainty about the standards of evaluation [21]. In the case we are discussing, Wikipedia editors may find it easier to agree on the quality of articles included in finer-grained knowledge categories [32]. The downgrading induced by difficulties in assessing quality of an item due to uncertainty about the appropriate evaluation schemas for the category to which the item belongs, is well documented in empirical studies of mediated markets such as, for example, the markets for financial services and entertainment products [9, 33, 34].

Basic level categories [35, 36], which are at an intermediate level between super-ordinate and sub-ordinate categories are claimed to be most useful for feature predictability [6]. This theory, transposed to Wikipedia, would predict that intermediate-level categories are the most useful and, hence, that granularity would relate in a curvilinear fashion with perceived quality—positively up to an ideal level and negatively thereafter. This prediction is somehow at odds with the principle of allocation claiming that the finer the category, the higher the evaluation.

One way to bridge this discrepancy is to take into account the positive relation between the amount of work invested in production and the expected quality of the resulting product. In the case of Wikipedia that we examine in this article, we expect that the number of edits received by articles will tend to increase the probability that these articles will be considered of high quality. Thus, two opposing “forces” pull the expected quality of articles in coarse-grained categories. On the one hand, they tend to attract a higher volume of editing activity which, in turn, may have positive implications for their expected quality. One the other hand, it is less likely that articles in coarse-grained categories will garner broad consensus and draw convergent evaluations from Wikipedia editors. Conversely, articles in fine-grained categories receive sharper categorical identity. Consequently, their quality is easier to evaluate within the narrower boundaries of their category. At the same time, articles in fine-grained categories tend to attract a lower volume of editing activity, which, in turn, is likely to have a negative effect on their perceived quality.

This reasoning may be summarized by the following expectations: the coarser is the granularity of a category, the higher will be the editing activity observed on the articles that it contains. Because granularity is measured in terms of path-distance from the root category (finer categories are more distant from the root and, thus, have higher granularity), *this prediction implies a **negative** relation between the granularity of a knowledge category and the tendency of articles that it contains to attract contributions*. Since sufficient amounts of contributions are expected to increase the quality of the article, *it follows as an indirect effect that there will be a **negative** relation between the granularity of a knowledge category and its perceived quality*. However, the coarser is the granularity of a category, the less likely articles contained in it will receive convergent evaluations of their quality. *This direct effect of granularity on quality implies a **positive** relation between the granularity of a knowledge category and the perceived quality of the articles that it contains*.

Clearly, these predictions only suggest qualitative associations among the variables of interest—rather than any specific functional form for the relation between granularity, volume of contribution, and perceived quality of the articles. In the empirical part of the paper we will let the analysis suggest more precise functional specifications that may imply non-linear functional forms—and hence go beyond the predictions of extant research on categorical identities that we have outlined.

We test these predictions using data that we have collected on articles in the English-language Wikipedia, the “free encyclopedia that anyone can edit” (en.wikipedia.org). Wikipedia is currently attracting considerable attention as an example of open knowledge production and organization system [37, 38], and as an empirical setting for studying how network structure affects knowledge creation [39], and knowledge search strategies [40].

We focus on the specific audience segment of Wikipedia editors (that is, users who contribute to an article), rather than the less circumscribed segment of Wikipedia readers. We do so because we are not interested in understanding what drives the general popularity of Wikipedia articles (i. e., “page views”), but rather in what determines their quality as evaluated by Wikipedia editors directly involved in the production process of Wikipedia articles. Our analytical focus is consistent with our view of Wikipedia not only as a knowledge repository, but also—and perhaps mainly—as a dynamic knowledge production system.

## Setting, data, and methods

### Data and variables

Traditionally studied within information and library science as an example of an ontological and lexical system [28], Wikipedia has recently been rediscovered as a collective production system of broader sociological interest [41].

Wikipedia is particularly useful to our current purpose because: (i) Classification of articles into predefined knowledge categories is transparent and based on a consistent and generally accepted classification hierarchy; (ii) the editing activity that an article attracts is directly observable; and (iii) publicly accessible quality evaluations produced by members of the Wikipedia community provide an unambiguous measure of quality that is generally understood and accepted within the community. Despite these apparently idiosyncratic features, Wikipedia is also: “The largest and most popular general reference work on the Internet and is ranked the fifth most-popular website” (https://en.wikipedia.org/wiki/Wikipedia). As such, Wikipedia shares many features with other knowledge artifacts openly produced and freely accessible through the Internet [42].

The empirical data that we analyze come from the database dump (https://dumps.wikimedia.org/) of the English-language version of Wikipedia from October 20, 2016. As units of analysis we consider all articles (i. e., Wikipedia pages in Namespace ‘0’, that are not redirects) that are members of at least one category, which are 5,006,601 out of 5,267,006 articles. For each article we define and compute the following variables as described below. The data are publicly available (https://doi.org/10.5281/zenodo.1005175).

#### Granularity

Wikipedia has two distinct types of categories: *content categories* (“intended as part of the encyclopedia, to help readers find articles, based on features of the subjects of those articles”) and *administrative categories* (“intended for use by editors or by automated tools, based on features of the current state of articles, or used to categorize non-article pages” https://en.wikipedia.org/wiki/Wikipedia:Categorization). We use the content categories to define the granularity of articles. In our data we have 1,060,885 content categories that contain at least one article. So far, categories of Wikipedia articles have been used in research mostly for building taxonomies [43, 44], rather than for understanding how social classification systems affect the production and evaluation of the knowledge they are designed to support and organize.

A category in Wikipedia may have an arbitrary number of parent categories and child categories. The directed category network, where links go from parent to child, has a dedicated root category which is Category:Articles. We define the *granularity* of a category *c* as the length of a shortest directed path from the root to *c* (see [10] for a similar strategy). Wikipedia articles may be members in multiple categories. The *granularity* of an article is defined as the average (mean) granularity of the categories of which it is a member. The distribution of the granularity of all articles is shown in S1 Fig (mean granularity over all articles is 7.59). Granularity of articles is used as an explanatory variable in our analyses, explaining the number of edits, the perceived importance, and the perceived quality of an article.

#### The *number of edits*

of an article is the number of times that a new version of the article has been uploaded. Note that the number of edits is, therefore, a direct measure of the volume of activity collectively produced by editors (i. e., the “producers”) of Wikipedia articles. As mentioned before, we are interested in article production and evaluation, rather than in the popularity of an article among readers (i. e., “consumers”) which might, for instance, be operationalized by the number of page views.

One of our predictions derived above is that the expected number of edits of an article decreases with its granularity. That is, articles in coarse-grained categories tend to receive more edits than articles in fine-grained categories. Another empirical regularity involving the number of edits is that the higher the number of edits of an article, the higher the probability that this article is perceived to be of high quality [45].

#### The *importance*

of an article is a binary variable that is equal to one if the article is labled “Top-importance”. Importance of articles in Wikipedia can be *Top*, *High*, *Mid*, or *Low*—roughly one third of articles have no importance label assigned (https://en.wikipedia.org/wiki/Category:Top-importance_articles). The decision whether an article has top importance is done by so-called WikiProjects. Thus an article might be of top-importance for one WikiProject but only of high or mid importance for another. (Note that this marks a difference to the featured article, or good article, status explained below which is a single label of excellence that applies for the whole of Wikipedia.) We say that an article is a top-importance article if it is so for at least one WikiProject. There are 37,594 top-importance articles in our dataset with the article Mathematics being one of them.

We expect that the probability that an article is labeled top-importance decreases with the granularity of its category. That is, articles in coarse-grained categories have a higher probability of being top-importance than articles in fine-grained categories. This prediction is consistent with the prediction that granularity decreases the number of edits: articles in coarse-grained categories are more likely to be perceived as important and editors tend to invest more work in them.

#### The perceived *quality*

of an article is also measured via a binary variable. We say that an article has high *quality* if it is a featured article (FA), see https://en.wikipedia.org/wiki/Wikipedia:Featured_articles. There are 4,848 FA, implying on average 9.7 FA per 10,000 articles. FA is the highest of Wikipedia’s seven assessment grades: featured articles, A-class, good articles, B-class, C-class, start-class, stub-class. Wikipedia’s article evaluations have been used in academic research before, e. g., in [46–48], and have been found to be consistent with external evaluations, e. g., in [46]. As a robustness check, we repeat some of our analyses with the variation that an article is defined to be of high quality if it is a featured or a good article (GA). There are about five times as many good articles as there are featured articles.

Several of our predictions have quality as the dependent variable. First, as noted above, we predict that the probability that an article is perceived to be of high quality increases with its number of edits. As indicated in the section “Theoretical background”, the relation between granularity and quality is expected to be more intricate. On the one hand, we derived from the principle of allocation that articles in fine-grained categories are more likely to be perceived of high quality so that the direct influence of granularity on quality is expected to be positive. On the other hand, we expect an indirect effect of granularity on quality via the number of edits as an intermediate variable that goes in the other direction: articles in fine-grained categories are expected to receive less edits and receiving less edits, in turn, is expected to lead to a lower quality. Thus, considering only the indirect effect via the number of edits, we expect that quality tends to decrease with granularity. Since it seems to be impossible to derive by theoretic arguments alone how these opposite effects interact, we will attempt to shed light on the three-way interplay between granularity, number of edits, and quality by a variety of non-parametric and parametric empirical analyses.

#### The *return on effort*

is a variable that measures the quality of the outcome relative to the work invested by Wikipedia editors. Here we take the point of view that the number of edits that an article receives may be seen as a measure of the collaborative effort produced (or cost borne) by Wikipedia editors in writing the article. This collective effort is rewarded (symbolically) if the article is featured. Formally, the *return* of an article is defined to be its quality (the boolean variable that is equal to one if the article is featured) divided by its number of edits. Thus the return of a featured article is one divided by the number of edits and the return is zero for a non-featured article. The measure gets more intuitive if we consider the average return of a set of articles which is the number of featured articles in this set divided by the cumulative number of edits performed to articles in it.

The expected link between granularity and return is clearer than the ambivalent expected relation between granularity and quality. From the principle of allocation we can derive that articles in coarse-grained categories are expected to need more work to achive high quality than articles in fine-grained categories so that return is expected to be positively related with granularity. That is, the average return of articles in coarse-grained categories is predicted to be lower than the average return of articles in fine-grained categories.

#### Top-level categories

An alternative explanation for the relations hypothesized above could be that articles in different topic areas have different latent probabilities of being featured, or tend to receive different number of edits, and that these topic areas accidentally happen to be assigned to knowledge categories with different levels of granularity. To show that findings are robust to these reasonable conjectures, we estimate models linking granularity to expected number of edits and to the probability to be featured separately for the 17 top-level categories (TLC) that are direct subcategories of either or both of Category:Main topic classifications or Category:Fundamental categories. To assign articles to TLC we first compute the distance (i. e., the length of a directed shortest path in the parent-child category network) from every TLC to every (non-TLC) category. A category is then assigned to all TLC to which it has the minimum distance. Articles, in turn, are assigned to the TLC of their categories. Thus, top-level categories seen as sets of articles can and do overlap. The top-level categories along with the number of articles in them are given in S3 Table.

#### Control variables

To further explore the robustness of the predicted relation between granularity and quality we include several other control variables that could (and, empirically, do) have an effect on the probability to be featured. These variables include the logarithm of the *length* (i. e., the number of bytes) of the article’s text; the article’s *age* (i. e., the time since the first edit); the logarithm of the *number of reverts* in its edit history; the logarithmized *number of contributors* (i. e., the number of unique users contributing at least one edit; we did not exclude anonymous users); two variables for the so-called reading complexity, namely the *average number of characters per word* and the *average number of words per sentence*; the numbers of *sections at level one* and *sections at level two*; the logarithm of the numbers of links of three different types, namely *intra-wiki links* pointing to another page in the English-language Wikipedia, *external references* pointing to Web-pages outside of Wikipedia, and *inter-language links* pointing to pages in another language edition of Wikipedia; the logarithm of the *number of images*; the logarithm of the *number of templates*; the *number of categories* in which the article is a member; and the *average size of categories* (the size of a category is the number of articles in it) in which the article is a member.

### Methods

We test our predictions with a combination of non-parametric and parametric statistical methods that have complementary advantages. The non-parametric methods make no assumption on the shape of the relation between variables. Thus, they are appropriate to assess agreement or disagreement of hypothesized relations (e. g., monotonic vs. curvilinear) with the empirical data. Moreover, visual non-parametric methods make the size of the effects more transparent. On the other hand, parametric methods can control for larger numbers of variables and yield a principled approach to assess the statistical significance of findings. The analysis has been done in R [49].

#### Non-parametric analysis

For the nonparametric analysis, we partition articles into 10 equally sized classes obtained by splitting at the deciles of the granularity values (ties are broken randomly to ensure that classes have the same size plus/minus one article). The deciles (plus the maximum value) of article granularity are shown in S1 Table. The classes obtained by that process are referred to as *granularity classes* and indexed from one to ten by increasing granularity, so that articles in finer categories are in classes with a higher index. Separately for the ten granularity classes we compute the average number of edits, the average probability that articles in the respective classes are featured, the average probability that articles in the respective classes are labeled top-importance, and the average return and display these values in bar charts.

By a similar process we partition articles into 10 equally sized classes (referred to as *edit classes*) obtained by splitting at the deciles of the number of edits (ties are broken randomly to ensure that classes have the same size plus/minus one article). The deciles (plus the maximum value) of the number of edits are shown in S2 Table. Separately for the ten edit classes we compute the probability that articles in the respective classes are featured and display these values in a bar chart.

Since it turns out (see the “Results” section) that articles in all but the highest edit class have a vanishing probability of being featured, we test the robustness of the relation between granularity and return restricted to articles in edit class 10. To ensure that we compare only articles with roughly the same number of edits we further sub-divide edit class 10 into 10 sub-classes defined by the number of edits. Thus, in a robustness check we consider the 10% of articles that have the highest number of edits (i. e., those 500,661 articles that have at least 165 edits). For this set we compute the deciles of the number of edits and the deciles of the granularity values, to compute the edit and granularity classes of this subset. We partition the subset into 100 classes where an article is put into class *c*_*ij*_, for *i*, *j* = 1, …, 10, if it is in edit class *i* and granularity class *j*. We compute the average return for each of these 100 classes and plot these values, grouped by edit class, in a bar chart.

#### Parametric analysis

For the parametric analysis we use two kinds of (generalized) linear regression models: linear regression specifying the logarithm of the number of edits as a function of the granularity of articles (in the parametric analysis we use the raw granularity values, rather than the indices of the granularity classes) and logistic regression specifying the probability that an article is featured as a function of the logarithm of the number of edits, the granularity and the squared granularity. In robustness checks we estimate logit models explaining the FA-probability separately for articles from the 17 top level categories and we extend the model by controlling for the other variables introduced above.

## Results

### Nonparametric analysis

**Fig 1:**
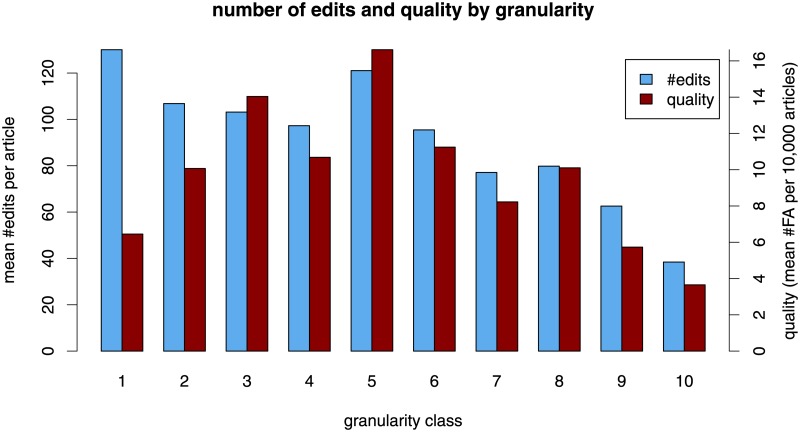
Number of edits and quality by category granularity. The noteworthy pattern is visible in the left-hand side of the diagram (articles in coarse categories). The 20% of articles with the coarsest categories receive above-average numbers of edits but their quality evaluations deteriorates compared to those with a medium category granularity.

Fig 1 (blue bars) displays the average number of edits of articles in the ten granularity classes. The diagram shows a clearly decreasing relation between granularity and number of edits (monotonicity, however, is broken at Class 5 and 8) implying that articles in coarser categories (left-hand side) tend to attract more edits. For instance, the 10% of articles with the lowest granularity (i. e., those in the coarsest categories) receive on average 130 edits, 42% more than the average over all 5 million articles (which is 91 edits) and 3.4 times the average number of edits of the most specific 10% of articles (38 edits).

**Fig 2:**
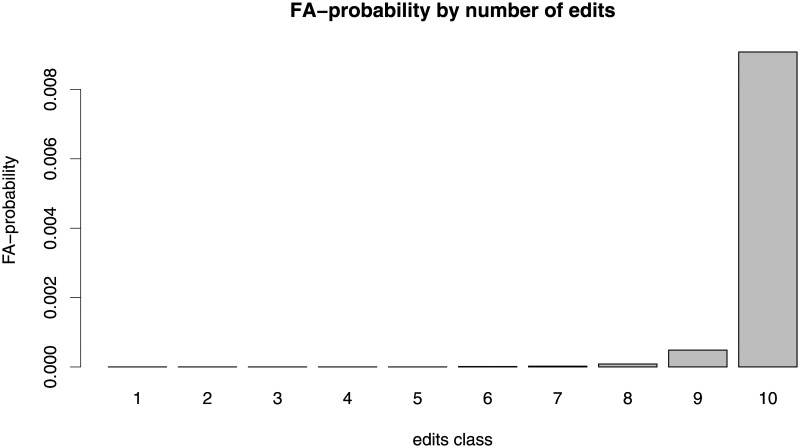
Average probability to be featured for articles in 10 edit classes.

Fig 2 displays the average probability to be featured for the ten edit classes. This probability increases sharply with increasing number of edits. There are zero FA in edit classes 1 through 5 (i. e., articles with less than 29 edits), five FA in edit class 6, 10 FA in edit class 7, 45 FA in edit class 8, 242 FA in edit class 9, and 4,546 FA in edit class 10. The 10% of articles with the highest number of edits (i. e., articles with at least 165 edits) have a rate of more than 90 FA per 10,000 articles (almost 10 times the overall rate). The 90% with the lowest number of edits have a rate of 0.67 FA per 10,000, more than a hundred times lower than the rate in edit class 10.

The relation between article granularity and the probability to be featured is displayed by the red bars in Fig 1. Combining the relation shown in the blue bars in Fig 1 (expected number of edits decreases with increasing granularity) and the relation shown in Fig 2 (FA-probability sharply increases with the number of edits), we could guess that the FA-probability decreases with increasing granularity. However, the red bars in Fig 1 show that this holds only in the right-hand side of the diagram (Classes 5 to 10) where the FA-probability varies in the same direction as the number of edits. Thus, in this interval, the more specific articles—typically receiving lower number of edits—also tend to receive lower quality ratings. This co-variation seems natural, since receiving sufficient work from editors is necessary for achieving high quality (compare Fig 2).

A striking empirical regularity is that the average number of edits and the FA-probability move in opposite directions for the articles in coarser-grained categories (left-hand side of Fig 1). For instance, the coarsest 10% of articles receive on average 129 edits (42% more than the overall average of 91 edits) but have a rate of only 6.6 featured articles per 10,000 (32% less than the overall rate of 9.7). These 10% of articles in the coarsest categories have a lower FA-probability than granularity Classes 2 through 8, all of which tend to receive lower numbers of edits. The average FA-probability of the coarsest 10% of articles is roughly comparable to that of granularity Class 9 which receives on average only half the number of edits of Class 1. Thus, the high number of edits garnered by articles in coarse-grained categories, tends to be associated with relatively unfavorable evaluations of their quality level.

**Fig 3:**
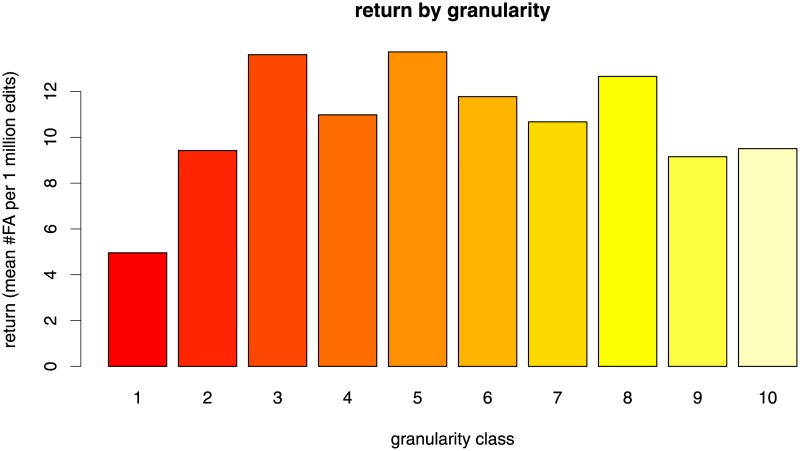
Average return for the ten granularity classes. Darker bars represent coarser articles.

The finding that articles in coarse-grained categories seem to be harder to write (they have lower appeal for potential contributors) becomes even more transparent if we look at the relation between granularity and return on effort. (Recall that average return of a set of articles is defined as the number of featured articles divided by the cumulative number of edits.) Fig 3 reveals that the return is relatively constant over the granularity classes except for the class containing the coarsest 10% of articles. The set containing the coarsest 10% of articles returns on average 5.16 FA for one million edits. The articles in granularity classes 3–8 all return more than 10 FA for one million edits. The 10% of articles with the lowest granularity (i. e., in the coarsest categories) tend to attract many edits, but the collective effort of editors is poorly rewarded in terms of quality.

**Fig 4:**
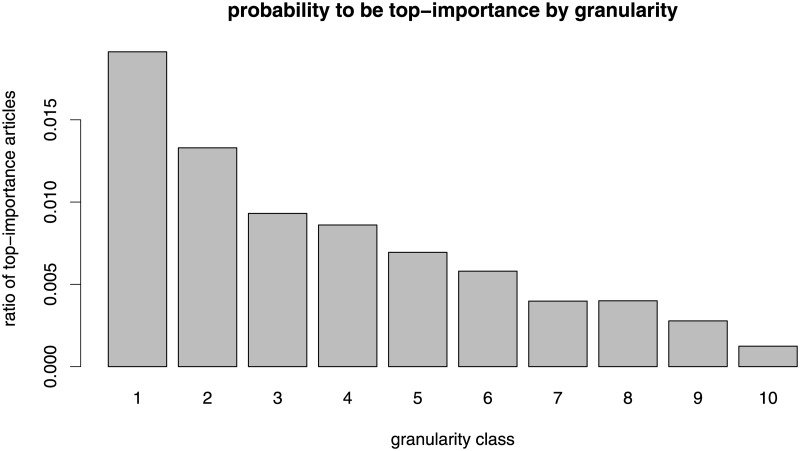
Probability of being top-importance for the ten granularity classes.

The combination of high numbers of edits and low probability to be featured—which occurs for the coarsest 10% of articles—is even more remarkable since articles in this set also have the highest probability to be labeled top-importance. Fig 4 displays the average probability of being a top-importance article for the ten granularity classes. There is a clearly monotonic decrease of importance with increasing granularity.

In summary, the coarsest 10% of articles not only tend to draw the highest volume of contributions by editors, but they also have the highest probability to be perceived as being of top importance—yet, they have a low probability to be awarded the featured article label. In contrast, the low quality of the 10% of articles in the most specific categories (highest granularity) might be explained by the low number of edits they tend to receive, that is, not enough work has been invested in them, and/or by the observation they are not perceived as important for the encyclopedia, that is, Wikipedians do not care enough about them.

**Fig 5:**
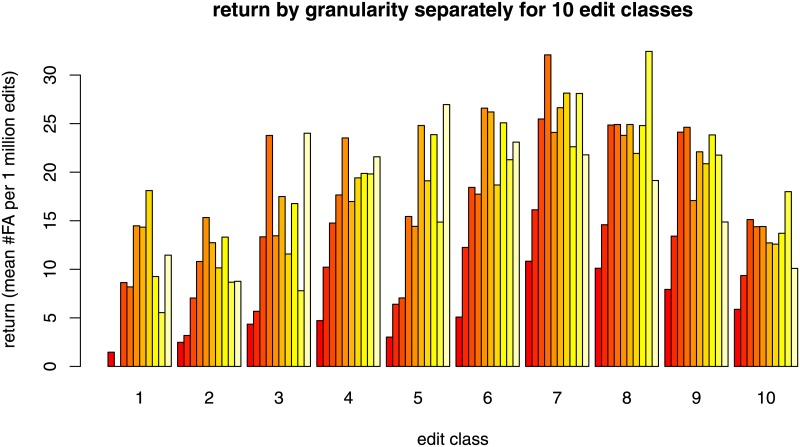
Return by category granularity separately for 10 different edit classes restricted to the 10% or articles receiving the highest number of edits. The bar chart for each edit class can be read in the same way as the single bar chart in Fig 3.

The finding that work invested in contributing to articles in coarse-grained knowledge categories is poorly rewarded becomes even more evident if we compare the average retun only for sets of articles with roughly the same number of edits, as described in the methods section above. Fig 5 shows consistently that articles in coarse-grained categories (dark red bars) are associated with lower returns in terms of quality ratings. The coarsest 20% of articles (dark red bars) consistently return the lowest number of FA per 1 million edits.

### Parametric models

The finding that articles belonging to coarser-grained categories are popular, but associated with lower return on editorial effort can be further examined with less descriptive parametric statistical models. Table 1 reports estimated parameters of a linear regression model with the logarithm of the number of edits as the response variable.

**Table 1:** Linear regression for the logarithm of the number of edits.

(Intercept)	5.063 (0.003)*
granularity	−0.218 (0.000)*
Adj. R^2^	0.059
Num. obs.	5,006,601
RMSE	1.299

* *p* < 0.001

The negative parameter associated with granularity indicates that articles in coarser categories receive higher number of edits (compare the decreasing height of the blue bars in Fig 1). The linear model predicts that the number of edits decreases by 28% whenever granularity increases by one standard deviation (standard deviation of granularity is 1.50).

Table 2 reports the coefficients of a logistic regression model for the probability that an article is featured. We fit a model that uses as explanatory variables the logarithm of the number of edits and granularity and another model that additionally uses the squared granularity.

**Table 2:** Logistic regression for FA-probability.

	w/o squared granularity	with squared granularity
(Intercept)	−14.719 (0.124)*	−21.906 (0.594)*
log(#edits)	1.310 (0.009)*	1.306 (0.009)*
granularity	0.189 (0.013)*	2.187 (0.162)*
squared.granularity		−0.136 (0.011)*
AIC	55,370.161	55,161.533
Num. obs.	5,006,601	5,006,601

* *p* < 0.001

The first model indicates that articles are more likely to be featured if they receive higher number of edits and more likely to be featured if they are in finer categories. The generalized linear model predicts that the odds of becoming featured gets multiplied by a factor of 5.78 whenever the logarithm of the number of edits increases by one standard deviation (*sd* = 1.34) and it predicts that the odds of becoming featured gets multiplied by a factor of 1.33 (i. e., increases by 33%) whenever the granularity increases by one standard deviation. The second model yields a negative coefficient for the squared granularity, which is consistent with the non-monotonic shape of the red bars in Fig 1 that takes a maximum for intermediate values.

We might still hypothesize that other characteristics of the articles determine the probability of being featured and accidentally correlate with granularity. To further test the robustness of our findings, we fit a more complex model for the probability of being featured (including article characteristics such as length, age, and numbers of editors, images, links, and external references—as described in the methods section) and show that it yields qualitatively the same results for the effects of the number of edits and granularity on article quality, see Table 3.

**Table 3:** Logistic regression for FA-probability.

	Model 1	Model 2
(Intercept)	−25.075 (0.393)*	−35.306 (0.782)*
log1p.length	2.377 (0.033)*	2.354 (0.033)*
age	0.903 (0.026)*	0.919 (0.026)*
log1p.#reverts	1.007 (0.024)*	1.037 (0.024)*
log1p.#contributors	−2.666 (0.047)*	−2.719 (0.047)*
#characters.per.word	−0.611 (0.024)*	−0.588 (0.024)*
#words.per.sentence	−0.186 (0.036)*	−0.183 (0.036)*
#level.1.sections	−0.312 (0.015)*	−0.320 (0.015)*
#level.2.sections	−0.466 (0.012)*	−0.458 (0.012)*
#categories	0.052 (0.006)*	0.036 (0.007)*
log1p.average.category.size	−0.045 (0.023)	−0.106 (0.023)*
log1p.#intra.wiki.links	−0.798 (0.031)*	−0.778 (0.031)*
log1p.#external.references	−0.126 (0.016)*	−0.119 (0.016)*
log1p.#inter.language.links	0.341 (0.020)*	0.329 (0.020)*
log1p.#images	0.152 (0.016)*	0.154 (0.016)*
log1p.#templates	0.686 (0.033)*	0.704 (0.033)*
**log1p.#edits**	**1.595** (**0.048**)*	**1.607** (**0.048**)*
**granularity**	**0.185** (**0.023**)*	**4.469** (**0.281**)*
granularity.squared		−0.440 (0.029)*
AIC	40,773.040	40,474.282
Num. obs.	4,983,893	4,983,893

* *p* < 0.001

All variables have been normalized to standard deviation equal to one before estimation. Variables with “log1p” in their name have been transformed by the mapping *x* ↦ log(1 + *x*) before normalization.

**Fig 6:**
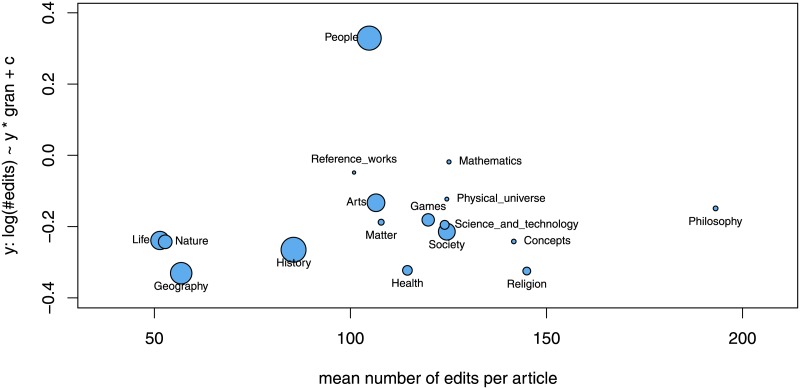
Mean number of edits and effect of granularity on the number of edits, separately for each TLC. Mean number of edits is displayed in the *x*-axis. The linear regression coefficient *α*_1_ of the granularity variable explaining the number of edits (compare Eq 1) is displayed in the *y*-axis. Area of points is proportional to the number of articles in the respective top-level category. All parameter estimates are significantly different from zero (*p* < 0.001).

An alternative explanation for the findings obtained so far could be that articles in different topic areas have different latent probabilities of being featured, or tend to receive different number of edits, and that these topic areas accidentally happen to be assigned to knowledge categories with different levels of granularity. Fig 6 displays, separately for all TLC, the mean number of edits in the TLC (*x*-axis) and the coefficient *α*_1_ from Eq (1)

$$\begin{array}{c} \log\left(\#edits\right)∼\alpha_{0}+\alpha_{1}\cdot granularity \end{array}$$ (1)

giving the slope of a linear approximation of the logarithmized number of edits by granularity (*y*-axis). All coefficients, except that for TLC “People”, are negative, implying that coarser categories do attract higher numbers of edits, supporting the aggregate finding obtained by analyzing all articles jointly.

**Fig 7:**
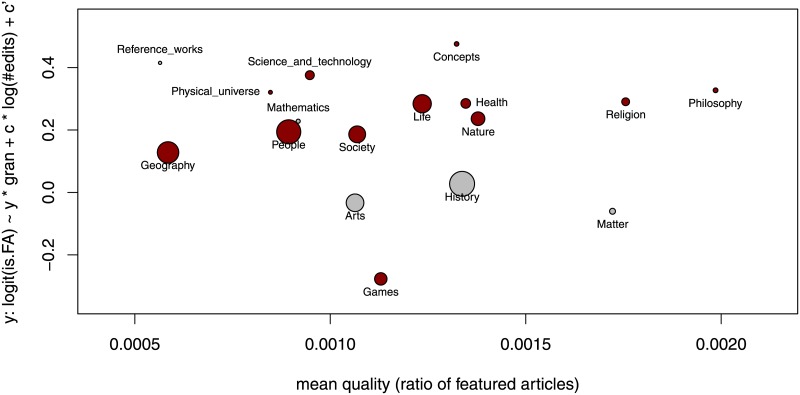
Average quality and coefficient of granularity explaining quality, separately for each TLC. The baseline probability of featured articles in the respective TLC is displayed in the *x*-axis. The logistic regression coefficient of the granularity variable, when controlling for the number of edits (parameter *θ*_2_ in Eq 2), is displayed in the *y*-axis. Coefficients that are significant (insignificant) at the 5% level are displayed as red (gray) dots.

Fig 7 displays for all TLC separately the average quality (that is, the rate of featured articles) in the respective TLC (*x*-axis) and the coefficient *θ*_2_ from Eq (2)

$$\begin{array}{c} \log(\frac{p}{1-p})∼\theta_{0}+\theta_{1}\cdot \log\left(\#edits\right)+\theta_{2}\cdot granularity \end{array}$$ (2)

—where we denote the probability that an article is featured by *p*—giving the slope of a linear approximation of the logit of the quality by granularity. Five of the 17 estimated coefficients *θ*_2_ are not significantly different from zero at the 5% level (the corresponding TLC are displayed as gray dots). These are the estimates for the three relatively small TLC *Mathematics*, *Matter*, and *Reference_works* and the estimates for the TLC *Arts* and *History* that are close to zero (these latter two coefficients are −0.033 and 0.027, respectively). Of the significant estimates for the remaining 12 TLC (displayed as red dots) all but one (associated with TLC “Games”) are positive, implying that more specific articles tend to be of higher quality when we control for the number of edits they receive. From another point of view, the coarser articles tend to get lower evaluations. This effect is reversed for TLC “Games.”

Yet another alternative explanation for the finding that more specific articles tend to be associated with a more positive social evaluation could be that Wikipedia articles become more precisely classified during the course of their development (and, naturally, they are more likely to become featured at later stages in their development, than while being still incomplete). However, this alternative hypothesis has to be rejected considering the relation between category granularity and the number of edits: the higher the number of edits of an article, the coarser (rather than finer!) are its associated categories (see the inverse of the relation displayed by the blue bars in Fig 1). Also note that controlling for article age, length, and many other characteristics that encode the state of development of an article does not change the link between granularity and quality, see Table 3. It is more plausible that the category granularity is exogenously determined by the article’s topic rather than its current state of development. For instance, the article Mathematics will always be a member of categories high up in the hierarchy—irrespective of its completeness or quality. Note however that the exceptional finding for the top-level category “People” in Fig 6 (in which articles that are more specific get more edits) suggests that the alternative hypothesis might be true for this particular topic area—but not for the whole of Wikipedia.

If, in a further robustness check, we weaken the criterion for being a high-quality article to those being featured or good articles, the shape of the relation between granularity and quality is nearly indistinguishable compared to that shown in Fig 1, where we considered only FA as high-quality articles. The relation between article granularity and this more inclusive measure of quality (see S2 Fig) does not change. In particular, we still find that coarser-grained articles have a relatively low probability to be of high quality. There are about six times as many articles that are featured or good, as there are featured articles.

Overall, the finding that articles in coarser knowledge categories garner a higher number of edits, but draw poor evaluations turns out to be quite robust to alternative explanations.

## Discussion and conclusions

We found that the structural position that articles occupy in the hierarchy of knowledge categories of the “free encyclopedia that anyone can edit” affects both the number of edits that articles are likely to receive, as well as the social evaluation of their quality.

More specifically, we found that articles in categories that are located closer to the top of the knowledge classification hierarchy (coarse-grained categories) tend to attract a higher number of edits (blue bars in Fig 1). To the extent that the volume of editing activity may be interpreted as a signal of attention, our analysis shows that Wikipedia editors pay more attention to articles in coarse-grained categories. Consistent with this result, articles in coarse-grained categories have the highest probability to be considered as being of “top-importance” (Fig 4). Yet, the articles in coarse-grained categories are less likely to reach high quality status as measured by the probability of being featured (red bars in Fig 1), leading to the seemingly counterintuitive finding that Wikipedia editors apparently find it problematic to write high quality articles about topics that are of particular interest to the Wikipedia community. The finding that articles in coarse-grained categories tend to receive a higher number of edits but tend to be of lower quality is even more remarkable since having a high number of edits turned out to be a strong positive predictor for article quality (compare Fig 2 and the positive coefficients associated with the logarithm of the number of edits in Table 2).

The analysis of the relation between granularity and quality gets complicated, to a certain extent, by the mediating variable *number of edits*. Articles that are located at the lower end of the granularity scale (articles included in coarse-grained categories) show different relations between the number of edits, perceived importance, and quality as compared to articles on the upper end of the granularity scale (articles in fine-grained categories). Articles in fine-grained categories receive relatively fewer edits, they are less likely to be perceived as important, and they tend to be of lower quality. These regularities seem to be intuitive and easily interpretable since articles in which little work is invested and which, in addition, are not perceived as important are likely to be incomplete and their quality is therefore likely to be discounted. At the same time, the low quality of articles in coarse-grained categories cannot be explained by insufficient effort by editors, or by being perceived as not important. In contrary, it seems that editors care most about these articles and they try to improve them by investing energies in considerable editing work. Yet, it seems that they do not succeed in bringing these articles in coarse-grained categories up to Wikipedia community standards. Thus, while articles in categories that are “too coarse” and articles in categories that are “too narrow” both tend to have inferior quality, the explanations for this finding are different for the two classes.

The second model in Table 2 also includes a square term for granularity to capture the potential non-linearity suggested by the non-parametric analysis in the relation between granularity and the probability that an article will be featured. According to the estimates reported in Table 2, the relation between granularity and the probability that an article will be featured turns from positive to negative when granularity exceeds the value of 8.04 (that is, the quadratic polynomial reaches its maximum at this granularity value). This value is located to the right of the median value of granularity observed in the sample (which is 7.44) and to the right of the mean granularity (which is 7.59). The granularity value where the maximum probability to be featured is assumed is in fact between the 70% and the 80% quantile. Articles with granularity equal to the “optimum”, thus, fall in granularity class 8 of 10 that have been defined in the section on data and methods. In other words, granularity of a category has to reach a fairly high level before further increases in granularity depress the probability that an article included in the category will be featured. We note that the “optimal” granularity value suggested by the non-parametric analysis in Fig 1 (red bars) is lower, since this non-parametric analysis does not control for the quality-increasing effect of the number of edits.

The finding that the articles in the coarsest categories show the seemingly paradoxical combination of high investment of work and low outcome quality gets clearer when we analyze the *return on effort*, which was defined as the number of featured articles divided by the cummulative number of edits. The non-parametric analysis displayed in Figs 3 and 5 showed consistently that articles in coarse-grained categories return less high-quality articles for a given amount of work. From another point of view, these articles need more work in order to achieve high quality than articles in fine-grained categories.

In our paper we focused on the specific audience segment of editors (i. e., users who contribute to articles), rather than the broader audience of readers [40], because our analysis treats Wikipedia as the outcome of a concrete knowledge production system [50]. As such, its most fundamental input—and the most stringent limit to its growth—is represented by the attention that editors allocate to the production of high quality ideas by editing Wikipedia articles. It is hard to think of a more important and vital set of questions than those pertaining to the factors that regulate the engagement of Wikipedia editors and the quality of their contribution.

Our analysis reveals a systematic tendency of coarse-grained articles to attract high levels of attention by editors, to be considered as important, but also to be considered of lower quality. How could this happen? How could it be that Wikipedia editors devalue the articles that they find more engaging?

The interpretation we offered rests on the assumption that Wikipedia articles compete for audience attention. The principle of allocation implies that editors can allocate only a finite amount of work to articles—and editors’ attention varies in part as a function of the granularity of the categories in which articles fall. A broader set of potential editors may be available to work on articles in coarse-grained categories because of their more permeable and lenient boundaries.

As an anecdotic illustration, consider the following case: a larger number of potential editors may be willing and able to contribute to “Mathematics” than to “Euclidean algorithm” as the term “Mathematics” might draw attention from a larger, and more diverse, set of potential editors. On the other hand, the term “Euclidean algorithm” is likely to be less accessible without the domain-specific knowledge of professional mathematicians. In consequence, we find that articles in coarse-grained categories tend to draw attention from a broader set of potential editors.

Our analysis also revealed a systematic tendency of articles in coarse-grained categories to attract lower quality ratings. Why does this happen? Building on the results of current research on critical social evaluation [51, 52], we have argued that Wikipedia editors will find it more difficult to evaluate the fit of articles to the more ambiguous boundaries of coarse-grained categories. This difficulty injects uncertainty into the evaluation process and may be responsible for the systematic quality discounts that empirical studies repeatedly document, and that our analysis so clearly reveals in the context of Wikipedia.

Returning to our running example, comparing aspects of the two Wikipedia articles “Mathematics” and “Euclidean algorithm”, the former states that “*There is a range of views among mathematicians and philosophers as to the exact scope and definition of mathematics*” (https://en.wikipedia.org/wiki/Mathematics accessed on July 11, 2017). Obviously, if it is not agreed upon what mathematics is, there will also be diverging views on what the Wikipedia article about it should contain. On the other hand, there seems to be little or no disagreement about what the Euclidean algorithm is. Consistent with this interpretation, the article on the Euclidean algorithm has been recognized as a featured article while the article on mathematics is only rated B-class.

The results we report are consistent with empirical studies that have found similar support for the principle of allocation in different contexts [7], and with theories of social categorization predicting that objects incorporating features from multiple categories will be considered less appealing than objects included in narrower, specialized categories [32, 53].

Our results also help us to go beyond existing studies arguing that objects with unclear classification, i. e., objects in coarse-grain categories tend to be ignored [9]. We found that this is not necessarily the case as the value of objects in coarse-grained categories may be discounted even when they attract above-average attention, and are perceived to be particularly important, from the relevant audiences.

How novel is this result and why should it matter? In the context of Wikipedia, this result begs explanation because the tendency of coarse-grained articles to be of low quality, despite the higher number of edits, may strike some as paradoxical—if not unusual. While it is natural to think of the quality of objects as dependent exclusively on internal properties of the objects themselves, the attention an object attracts and how its value is assessed by relevant audiences are frequently based on categorical judgments, i. e., judgments that depend on the position that an object occupies in a space of categories [3]. The existence of reference-dependent judgment is not new in itself [54, 55], but we are not aware of work that has documented its effects at a comparably global scale and in non-experimental empirical settings.

Similarly well understood are the effects on audience appeal and social evaluation of the sharpness of categorical boundaries—or “contrast” [56]—and the ambiguity of categorical boundaries—or “leniency” [5]. Unlike results reported by contemporary studies developing this line of research, however, the results of the present study are based on a definition of categorical boundaries that is independent of the grade of membership of articles in knowledge categories. In the case we have examined, categorical boundaries depend only on the position that a category occupies within a hierarchical classification system that is exogenous with respect to short-term variations in the set of articles—unlike the majority of empirical cases of commercial products and services studied so far.

## Supporting information

S1 Fig Granularity distribution. Number of articles by granularity.
(EPS)

S2 Fig Considering also good articles as high-quality. (EPS)

S1 Table Deciles of article granularity. (PDF)

S2 Table Deciles of number of edits of articles. (PDF)

S3 Table Top-level categories and numbers of articles in them. (PDF)
