# Supplementary material for: Knowledge categorization affects popularity and quality of Wikipedia articles
Source: PLoS One. 2018 Jan 2;13(1):e0190674. doi: 10.1371/journal.pone.0190674 (PMC5749832; doi:10.1371/journal.pone.0190674)
Supplement: S1 Table — (PDF) [file pone.0190674.s003.pdf]

S1 Table Deciles of article granularity.

10%	20%	30%	40%	50%	60%	70%	80%	90%	100%
6.00	6.50	6.90	7.10	7.44	7.75	8.00	8.50	9.00	19.50
