# Supplementary material for: Knowledge categorization affects popularity and quality of Wikipedia articles
Source: PLoS One. 2018 Jan 2;13(1):e0190674. doi: 10.1371/journal.pone.0190674 (PMC5749832; doi:10.1371/journal.pone.0190674)
Supplement: S2 Table — (PDF) [file pone.0190674.s004.pdf]

S2 Table Deciles of number of edits of articles.

10%	20%	30%	40%	50%	60%	70%	80%	90%	100%
6	10	15	21	29	39	55	84	165	46,075
