# Supplementary material for: Knowledge categorization affects popularity and quality of Wikipedia articles
Source: PLoS One. 2018 Jan 2;13(1):e0190674. doi: 10.1371/journal.pone.0190674 (PMC5749832; doi:10.1371/journal.pone.0190674)
Supplement: S3 Table — (PDF) [file pone.0190674.s005.pdf]

S3 Table Top-level categories and numbers of articles in them.

Arts	Concepts	Games	Geography	Health
748,905	49,132	371,124	1,107,673	222,769
History	Life	Mathematics	Matter	Nature
1,501,421	801,613	40,307	84,197	448,368
People	Philosophy	Physical_universe	Reference_works	Religion
1,396,225	53,898	35,418	24,805	145,816
Science_and_technology	Society			
191,045	681,301			
